# Supplementary material for: The effect of light conditions on the content of selected active ingredients in anatomical parts of the oyster mushroom (Pleurotus ostreatus L.)
Source: PLoS One. 2022 Jan 4;17(1):e0262279. doi: 10.1371/journal.pone.0262279 (PMC8726465; doi:10.1371/journal.pone.0262279)
Supplement: Attachment — Submitted filename: Response to Reviewers.docx [file pone.0262279.s001.docx]

5.11.2021

Dear Editiors and Reviewers,

Thank you for your thorough review. All comments and recommendations indicated by Reviewers have been included in the revised version of the manuscript. Changes to the manuscript were made in the content of the publication. The comments listed in the reviews are referenced in below.

**Response to the editor:**

1.	As the study mainly focusses on the effect of light on active ingredients on different parts of the oyster mushroom, thus the title may be revised accordingly.

**The title has been revised, and changed to: “The effect of light conditions on the content of selected active ingredients in anatomical parts of the oyster mushroom (Pleurotus ostreatus L.)”**

2.	The language in the abstract is too complicated using multiple statements in a single sentence. Thus the syntax of the sentences must be taken care off.

**The syntax was improved and sentences shortened.**

3.	Introduction part is too lengthy, may be reduced/limited to relevant statements with regard to the present study.

**The introduction part has been shortened.**

4.	In all over the manuscript, long sentences are used, which may be revised and short and clear sentences may be used.

5.	In introduction word variety is used in context of oyster mushroom, I am not sure whether it is variety of the same species or different species.

**It has been corrected or these parts has been deleted.**

6.	Careful Language correction is required in the manuscript.

**Language correction across manuscript has been conducted.**

**Response to the Reviewer no. 1**

Reviewer #1:

1. Line no. 63, "Winter varieties require temperatures below 15ºC". Is it is winter varities or winter species?

2. Line no. 63 Check sign of degrees and correct.

3. Line no. 64 Check sign of degrees and correct.

4. Line no. 66 Check sign of degrees and correct.

**This issues have been resolved.**

5. Line 76-78 need refernce to support

**Reference has been added.**

6. Authors can specify that why some particular vitamins are taken in study, if any particular reason for that?

**Such vitamins are the most present in oyster mushrooms**.

7. Improve discussion part

**Discussion has been improved.**

8. Mention the source and colour of light used in experiment

**This information has been added in the “material” section.**

**Response to the Reviewer no. 2**

Reviewer #2:

This manuscript tries to identify effect of cultivation conditions on the content of selected active ingredients in parts of the oyster mushroom that would help to develop nutritious and healthy mushroom production. This study is preliminary conventional because there have been larger trials that have shown similar findings. There are few typographical errors in the text, that must be corrected before the paper is accepted.

P1, L 21: Typographical errors

P4, L 92: Typographical errors

P9, L 207-208: Typographical errors

P10, L 220: Typographical errors

P11, L 237: Typographical errors

P12, L 239; Table 4: Typographical errors

P14, L 290: Typographical errors

P16, L 324, 325: Typographical errors

**Typographical errors have been corrected.**

We hope that the current state of the manuscript meets the standards of the Journal and that publication will be possible

Best regards,
Marcin Dziedziński
